# Crystal Structure of *Schizosaccharomyces pombe* Rho1 Reveals Its Evolutionary Relationship with Other Rho GTPases

**DOI:** 10.3390/biology11111627

**Published:** 2022-11-07

**Authors:** Qingqing Huang, Jiarong Xie, Jayaraman Seetharaman

**Affiliations:** 1 Department of Biological Sciences, 14 Science Drive 4, National University of Singapore, Singapore 117543, Singapore; 2 Department of Structural Biology, St. Jude Children’s Research Hospital, Memphis, TN 38105, USA

**Keywords:** GTPase, Rho, GDP, structure, evolution

## Abstract

**Simple Summary:**

Rho family of proteins are involved in cytoskeletal organization, cell mobility and polarity, and are implicated in cancer morphogenesis. The structure and function of the Rho homologs from higher-level organisms are well studied, but not from the lower-level organisms. Such as over 95% of the known structures of Rho GTPases are from higher-order mammalian organisms, with only three structures of Rho homologs reported to date from lower-level, single-celled organisms. In this paper we report the crystal structure of Rho1 from *Schizosaccharomyces pombe,* also called fission yeast (*Sp*Rho1), in complex with GDP in the presence of Mg^2+^ at 2.63-Å resolution, to broaden our understanding of Rho homologs in lower-level organisms. Although the overall structure is similar to that of known Rho homologs, we observed subtle differences at the Switch I and II regions, in β2 and β3, and in the Rho insert domain and loop from Phe107 to Pro112. Combined with literature and sequence analyses, we suggest that the Switch regions and Rho insert domain may contribute to downstream kinase activation in different species through their interactions with different effectors and regulators; and the conservation and divergence of Rho GTPases among difference species and provide evolutionary insight for *Sp*Rho1. While many studies have reported the evolutionary development of Rho GTPases based on their amino acid sequences, the present study, for the first time, explores these evolutionary aspects based on structure. Our analysis indicates that *Sp*Rho is evolutionarily closer to *Hs*RhoC than *Hs*RhoA, as previously believed.

**Abstract:**

The Rho protein, a homolog of Ras, is a member of the Ras superfamily of small GTPases. Rho family proteins are involved in cytoskeletal organization, cell mobility, and polarity, and are implicated in cancer morphogenesis. Although Rho homologs from higher-order mammalian organisms are well studied, there are few studies examining Rho proteins in lower-level single-celled organisms. Here, we report on the crystal structure of Rho1 from *Schizosaccharomyces pombe* (*Sp*Rho1) in complex with GDP in the presence of Mg^2+^ at a 2.78 Å resolution. The overall structure is similar to that of known Rho homologs, including human RhoA, human RhoC, and *Aspergillus fumigatus* Rho1 (*Af*Rho1), with some exceptions. We observed subtle differences at the Switch I and II regions, in β2 and β3, and in the Rho insert domain and loop from Phe107 to Pro112. Our analysis suggests that *Sp*Rho is evolutionarily closer to *Hs*RhoC than *Hs*RhoA, as previously believed.

## 1. Introduction

Hundreds of GTPases have been found in eukaryotic cells, playing various roles in signal transduction to control gene transcription, cell growth, and development. Small GTPases are a group of low-molecular-weight (20–25 kDa) guanine-nucleotide-binding proteins [1]. Most small GTPases comprise a conserved sequence of 5G boxes that are responsible for the GTPase activity and GTP/GDP binding [2]. Small GTPases can be divided into five families: Ras, Rho, Rab, Arf, and Ran. The Rho family was delineated from the rest because of its unique insert region between the conserved G4 and G5 boxes, which is absent among other GTPase families. Like other Ras-like GTPases, Rho GTPases can switch between inactive GDP-bound and active GTP-bound forms through the actions of guanine nucleotide exchange factors (GEFs), GTPase-activating proteins (GAPs), and guanine nucleotide dissociation inhibitors (GDIs) [3]. GEFs are usually bound with inactive GDP-bound Rho and require the exchange of GDP with GTP for activation. The active GTP-Rho, subsequently, hydrolyzes GTP to GDP intrinsically, also in response to GAP stimulation. Finally, GDIs bind Rho to prohibit the actions of GEFs or GAPs to positively or negatively regulate Rho activity, respectively, as required within the cell [1,4,5].

The Rho family is divided into nine subfamilies, Rho, Rac, Cdc42, RhoDF, Rnd, RhoUV, RhoH, RhoBTB, and Miro, with various differences in sequence, structural motif, and function [1]. These Rho subfamily proteins are known to influence cell morphogenesis, mobility, adhesion, migration, and changes in polarity [6] by controlling the assembly, regulation, and reorganization of the actin cytoskeleton [7,8]. Different species have different isoforms of Rho family proteins, three of which are well studied in mammalian systems: RhoA, B, and C. Much less is known about Rho homologs from lower-level eukaryotes, such as yeast, which comprises a subfamily of five proteins: Rho1, -2, -3, -4, and -5.

Various structures of Rho GTPases have been reported on in complex with GDP/GTP analogs or effector proteins [9,10,11,12,13]. Over 95% of the known structures of Rho GTPases is from higher-order mammalian organisms, with only three structures of Rho homolog reported to date from lower-level, single-celled organisms. In this study, we report on the crystal structure of Rho1 from *Schizosaccharomyces pombe* (*Sp*Rho1), also called fission yeast, to broaden our understanding of Rho homologs in lower-level organisms. Sequence and structural analyses illustrate the conservation and divergence of Rho GTPases among different species and provide evolutionary insight for *Sp*Rho1. While many studies have reported on the evolutionary development of Rho GTPases based on their amino acid sequences, the present study, for the first time, explores these evolutionary aspects based on structure.

## 2. Results

### 2.1. Structure of SpRho1–GDP Complex

**Figure 1:**
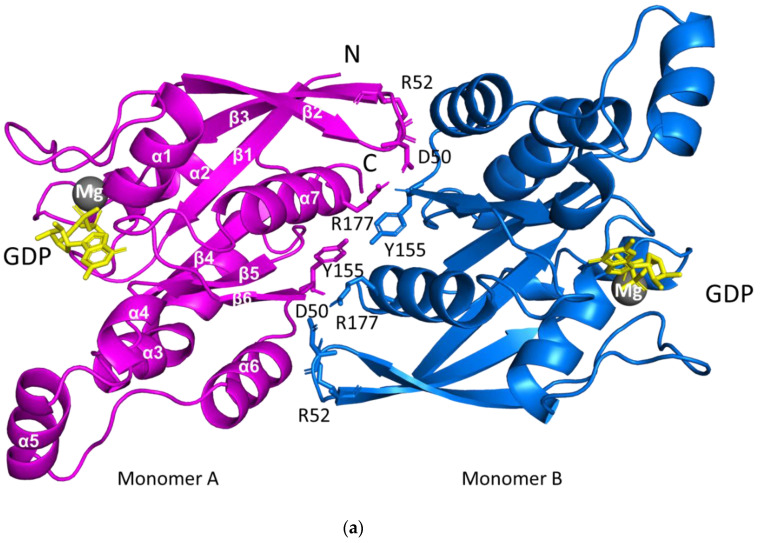

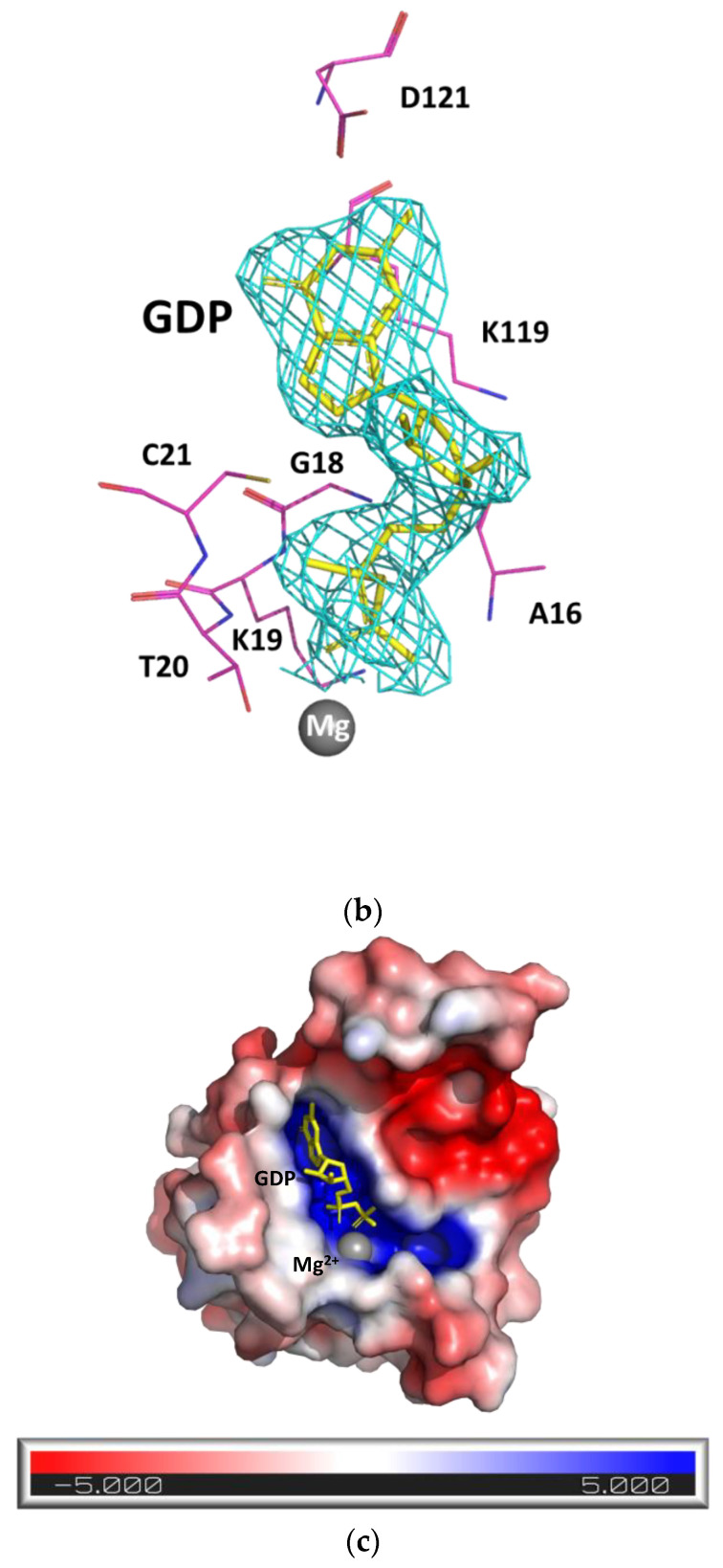
*Sp*Rho1–GDP–Mg^2+^ structure. (**a**) The ribbon diagram of the *Sp*Rho1–GDP–Mg^2+^ complex dimer. The GDP molecule is in yellow and Mg^2+^ is shown as a grey sphere. The key residues at the dimer interface are shown and labelled. The secondary structures are labelled on one monomer. (**b**) The *2Fo-Fc* electron density around GDP with 1 sigma contour level. The key residues of the GDP binding pocket are shown. (**c**) Electrostatic surface potentials are colored red and blue for negative and positive charges, respectively, and white color represents neutral residues. The GDP is bound in the highly positively charged pocket. All the structurally related figures in this paper were prepared using PyMOL (The PyMOL Molecular Graphics System, Version 1.2r3pre, Schrödinger, LLC.,New York, NY, USA).

The *Sp*Rho1–GDP complex structure was determined at a 2.78 Å resolution (Table 1). The gel filtration showed that the protein existed as a monomer, but the asymmetric unit comprised a dimeric *Sp*Rho1–GDP complex, which suggested the possibility of a monomer–dimer equilibrium in the solution. Monomers comprised of residues from Arg6 to Leu181 that were shown to be well defined in the electron density map. Each monomer of the dimer consisted of seven α-helices and a mixed twisted β-sheet consisting of six β-strands, of which five were parallel and one (the second) was antiparallel (Figure 1a). The *Sp*Rho1 dimeric interface incorporated α7, the turn region between β2 and β3, with a total buried area of 1495 Å^2^. The dimer was stabilized by several hydrogen-bonding contacts and hydrophobic interactions, with residues of Asp50, Arg52, Tyr155, and Arg177 from both monomers involved in hydrogen bonding (Table S1).

The bound GDP molecule was well defined in the electron density map and located within a positively charged pocket (Figure 1b,c) formed by residues Ala16, Gly18, Lys19, Thr20, Cys21, Lys119, and Asp121, along with a Mg^2+^ ion. The Mg^2+^ ion coordinated with residues Thr20 and Thr38, the GDP β-phosphorous oxygen and water molecules. The structure of the GDP-bound form of *Sp*Rho1 was similar to that of *Hs*RhoA–GDP–Mg^2+^ (*Hs: Homo sapiens*), which also showed a Mg^2+^ coordination with two threonine, GDP, and water molecules.

### 2.2. Comparison of Rho Subfamily Protein Structures

Despite there being numerous structures of Rho subfamily proteins in the PDB database, only three Rho structures were reported on in complex with GDP and a Mg^2+^ ion (*Hs*RhoA, *Hs*RhoC, and *Af*Rho1), with most structures shown in complex with effectors such as GDI or active GTP analogs, exhibiting conformational differences in the Switch I and II regions [9,14,15]. Since Mg^2+^ was known to affect the conformation of the Switch I region [16], we only compared our *Sp*Rho1–GDP–Mg^2+^ structure with the *Hs*RhoA–GDP, *Hs*RhoC–GDP, and *Af*Rho1–GDP complexes also containing Mg^2+^ (Table S2).

We found that *Sp*Rho1 resembled all three of the complexes examined, with good superimposition and RMSD values of less than 1 Å (Figure S1A). However, discrepancies were observed in five different regions (RMSD plot, Figure S1B), with subtle differences identified in the Switch I (Gly29–Ala45) and Switch II (Asp60–Val80) regions, the Rho insert domain, and at the loop region from Phe107 to Pro112. The fifth difference was observed only between *Hs*RhoA and *Sp*Rho1 in the β2 and β3 (Asn42–Asp60) region, with β2 and β3 of *Hs*RhoA positioned further outward than that in *Sp*Rho1.

### 2.3. Sequence Comparison of Rho Subfamily Proteins

Rho family members share 30% identity with other Ras GTPases, and 40% to 80% sequence identity within the Rho family [1]. Sequence identity is even more conserved among the Rho subfamily members, with approximately 85% identity shared among *Hs*RhoA, *Hs*RhoB, and *Hs*RhoC [8]. As would be expected, *Sp*Rho1 shares a much greater sequence identity to homologs of lower-level organisms than mammalian organisms.

**Figure 2:**
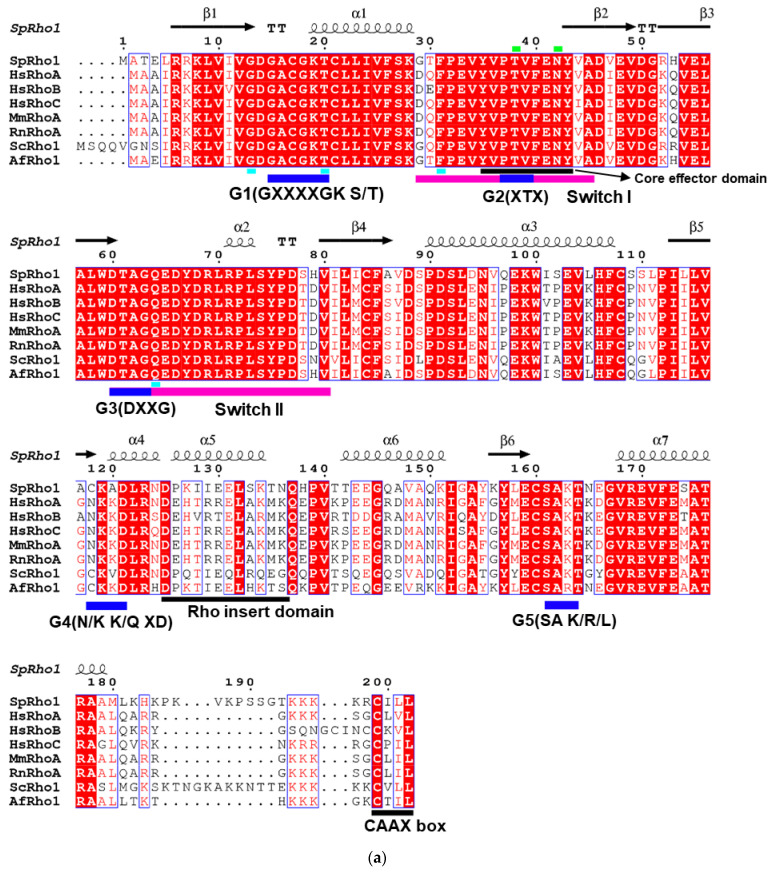

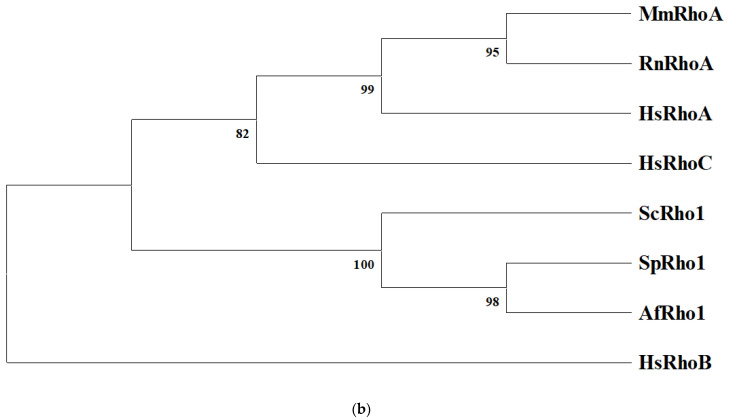
Sequence alignment and phylogenetic tree of *Sp*Rho1. (**a**) Sequence alignment of *Sp*Rho1, HsRhoA, HsRhoB, HsRhoC, MmRhoA, RnRhoA, ScRho1, and AfRho1. These sequences were selected corresponding to Supplementary Table S2. The secondary structure of *Sp*Rho1 is shown on the top. The blue bars represent the 5 conserved G boxes with specific sequences shown. The cyan bars (Gly13, Thr19, Phe30, and Gln63) indicate the important sites for GTP hydrolysis. Switch I and II regions are indicated with magenta bars. Core effector domain, Rho insert domain, and CAAX box are labelled with black bars. Blue frames indicate conserved residues. White letters in red boxes refer to strict identity, but red letters in white boxes indicate similarity. The amino acid sequences were aligned using Clustal Omega and Escript [25,26]. (**b**) Phylogenetic tree of *Sp*Rho1, HsRhoA, HsRhoB, HsRhoC, MmRhoA, RnRhoA, ScRho1, and AfRho1. It shows how species evolved from common ancestors and shows which are more related when they have a more recent common ancestor. The value at node indicates the frequency, which is the confidence of obtaining the same results using other programs. This reflects how species or other groups evolved from a series of common ancestors. Two species MEGA 11 software were used for preparing this tree [27].

Sequence alignment showed several conserved regions between *Sp*Rho1 and its homologs (Figure 2a). Specifically, the five G boxes are highly conserved and are involved in the exchange of GDP/GTP, GTP hydrolysis, and GTP-induced conformational change [2,8]. Wheeler and Ridley [17] identified the functional importance of Gly13, Thr20, Phe31, and Gln64 in the GTP hydrolysis of human Rho GTPases; we found that these residues were retained across all species, including yeast (Figure 2a). In addition, despite significant sequence conservation within the Switch I and Switch II regions, subtle conformational differences were observed for the inactive GDP-bound state (Figure S1A). These switches are turns between α-helices and β-strands, and serve as platforms for selective interactions with downstream factors to initiate cytoplasmic or nuclear signaling cascades [18,19]. Collectively, these observed structural differences may suggest different downstream activations among different species.

Apart from residues Asp125 and Leu132, the Rho insert domain (Asp125-Asn136) differs among species, with structural comparisons highlighting subtle differences within this region. The Rho insert domain is a signaling element found only in Rho family proteins, which reportedly has an impact on NADPH oxidase activation, Ras-induced apoptosis suppression, Rho kinase activation, and downstream cellular transformation [20,21,22,23].

Finally, there were clear differences between the yeast and mammalian proteins. The yeast Rho1 proteins (*Sp*Rho1 and *Sc*Rho1) had 8 aa longer C-terminal tails (Pro185-Gly192) than their mammalian counterparts (Figure 2a), which may have a role in membrane localization [24]. Indeed, two signals are required for membrane localization: the CAAX box and a polybasic or polycysteine domain upstream of the prenylcysteine residue that is used for palmitoylation [23,24]. Additionally, compared with mammalian Rho GTPases, yeast Rho GTPases also have an abundance of hydrophilic amino acids, such as serine or threonine, and the presence of these residues may influence the differences in the localization patterns of mammalian and yeast Rho GTPases.

### 2.4. Phylogenetic Analysis of Rho Subfamily Proteins

In exploring the evolutionary relationships between yeast and mammalian Rho proteins, we performed a phylogenetic tree analysis. We found a very early divergence of *Hs*RhoB from the other Rho proteins, as well as a clustering of Rho1 proteins from lower-level, single-celled organisms, which may indicate a common ancestor (Figure 2b). Yeast *Sp*Rho1 was evolutionarily closer to *Af*Rho1 than *Sc*Rho1 from yeast *Saccharomyces cerevisiae,* because it shared a more recent common ancestor with *Af*Rho1.

## 3. Discussion

Rho GTPases play essential roles in cellular development and signaling transduction. Previous structure/function analyses were primarily conducted using prototypical mammalian Rho GTPases, such as Rac1, RhoA, and Cdc42 [1,13,28,29]. Indeed, human RhoA is the most well-studied isoform from the Rho subfamily. RhoA shows extensive homology with other isoforms, such as RhoB or RhoC, with all three homologs playing similar functions in actin polymerization and stress fiber induction [17]. Numerous structures for human GDP/GTP-bound Rho proteins have been reported on, particularly RhoA in complex with binding effectors (Table S2), albeit structural analyses for RhoB and RhoC are limited. The mouse Rho homolog has also been well studied compared to yeast and fungus homologs, whose investigations are highly limited. Thus, an understanding of the present structure of Rho1 from *S. pombe* is crucial in filling the gaps in our understanding of the evolutionary relationships of RhoA subfamily proteins from different species, particularly for lower-level organisms such as yeast and fungus.

Based on our structure–sequence relationships, we suggest that *Sp*Rho1 may be evolutionarily closer to *Hs*RhoC than to *Hs*RhoA, as previously believed. Studies have shown that mammalian RhoA can substitute for *S. cerevisiae* Rho1 and confer full function in yeast cells [30]. The phylogenetic tree analysis, as predicted from amino acid sequences, indicated that *Hs*RhoA and *Hs*RhoC may have diverged at the same point from a Rho homolog from a single-celled organism (Figure 2a), suggesting a common ancestor for *Hs*RhoA, *Hs*RhoC, and *Sp*Rho1. As such, it is uncertain whether *Hs*RhoA is evolutionarily closer than *Hs*RhoC. The sequence analysis showed that *Sp*Rho1 and *Hs*RhoC had a 70% similarity, which was higher than that for *Hs*RhoA with *Sp*Rho1 (67%), albeit not significantly. Furthermore, the structure superimposition showed that 18 more Cα atoms of *Hs*RhoC could be superimposed to *Sp*Rho1 compared with *Hs*RhoA. These findings together may indicate that *Hs*RhoC is evolutionarily closer to *Sp*Rho1 than *Hs*RhoA is. However, this assumption should be taken with caution as we could not exclude *Hs*RhoB to be less related, because it branched off earlier in evolutionary history; indeed, *Hs*RhoB shared a high sequence similarity with *Sp*Rho1 (Figure 2b).

In conclusion, here, we determined and characterized the structure of *Sp*Rho1 and highlighted its similarities with other reported Rho subfamily structures. Our analysis of the unique and conserved features of Rho homologs suggested that *Sp*Rho1 is likely to be evolutionarily closer to *Hs*RhoC than *Hs*RhoA, and further predicted that the Rho insert region of the Rho GTPase likely contributes to the downstream kinase activation among different species; however, this warrants further studies. Our study offered further insight into the Rho protein family of single-celled organisms and the evolutionary conservation of this key protein compared with mammalian types, filling the gap in our understanding of the structure and function of RhoA proteins from lower-level organisms.

## 4. Methods and Materials

### 4.1. Protein Expression and Purification

BamHI and SalI sites were used for the cloning of the *Schizosaccharomyces pombe* Rho1 (*Sp*Rho1) gene (NCBI gene ID: 2541612) in the modified pET32b vector. The protein was fused with the N-terminal (His)6 tag and expressed in the *Escherichia coli* BL21 strain. BL21 cells were cultured in LB broth medium with 100 µg/mL ampicillin at 37 °C, and 150 µM IPTG was added for induction. Cells were harvested with centrifugation at 3500 rpm (JLA-8.1 rotor) for 30 min at 4 °C. Cell pellets were lysed in a lysis buffer (50 mM Tris-HCl at pH7.4, 200 mM NaCl, 5% *v*/*v* glycerol, 5 mM imidazole, 0.1% *v*/*v* Triton x-100, a protease inhibitor, and 5 mM β-mercaptoethanol (BME)), and then sonicated. The lysate was then centrifuged and the supernatant was added to the Ni-NTA resin that was equilibrated with a lysis buffer for 1 hr at 4°C. Next, it was washed with a wash buffer (50 mM Tris-HCl (pH7.4), 200 mM NaCl, 5% *v*/*v* glycerol, 10 mM imidazole, and 5 mM 2-mercaptoethanol (BME)) twice, and then eluted with an elution buffer (the elution buffer contained 50 mM Tris-HCl (pH7.4), 200 mM NaCl, 5% *v*/*v* glycerol, 400 mM imidazole, and 5 mM 2-mercaptoethanol (BME)). The eluate was further purified using a Superdex 200 column (GE Healthcare, Uppsala, Sweden) with a buffer containing 20 mM Tris-HCl (pH7.4), 100 mM NaCl, 5% *v*/*v* glycerol, 5 mM MgCl_2_, and 2 mM DTT. The purity was verified using 12.5% SDS-PAGE gel.

### 4.2. Crystallization and Structure Determination

The purified *Sp*Rho1 was concentrated using 10 kDa MW cut-off centricon (vivaspin) to 6 mg/mL. GDP was added to the *Sp*Rho1 protein to reach a molar ratio of 1:10 and incubated for 5 min on ice. The complex was crystallized using the hanging drop vapor diffusion method at room temperature (24 °C) with commercial crystallization screening kits such as Crystal Screen 1 and 2, Salt Rx 1 and 2, and Index^TM^ (Hampton Research), with a drop size 1:1 (µL) ratio of protein solution to reservoir solution. After the optimization of the initial conditions, diffraction quality crystals were obtained from a condition containing 0.2 M potassium sodium tartrate tetrahydrate, 0.1 M sodium citrate tribasic tetrahydrate (pH 5.6), and 1.9 M ammonium sulfate. Prior to data collection, the crystals were cryoprotected using the crystallization solution supplemented with 25% glycerol. The crystal diffracted up to 2.63 Å and data were collected using Rigaku MicroMax™-007 HF. The data were processed and scaled using HKL2000 [31]. There were two complex molecules in the asymmetric unit. PHENIX AUTO-MR [32] was used for structure determination using the PDB 1FTN as a search model for the molecular replacement method (Table S1). Model building was performed using COOT [33]. The final model had good stereochemical parameters, which were evaluated with PROCHECK [34].

## Figures and Tables

**Table 1:** Crystallographic data and refinement statistics for *Sp*Rho1–GDP complex with Mg^2+^.

Unit Cell Parameters (Å, ◦)	a = 105.69, b = 66.35, c = 75.55, α = γ = 90, β = 112.78
Space group	C2
Data collection	
Resolution range (Å)	50.00–2.78 (2.83–2.78) *
Wavelength (Å)	1.5418
Unique reflections	12485 (617)
Completeness (%) Redundancy	100.0 (99.8) 4.4 (4.4)
Overall (I/σ(I))	3.3 (2.8)
R_sym_^a^ CC ^1/2^ CC *	19.0 (0.818) 0.952 (0.518) 0.988 (0.824)
Refinement and quality ^b^	
Resolution range (Å)	50–2.78
R_work_^c^	0.19
R_free_^d^	0.23
Root mean square deviation	
Bond length (Å)	0.010
Bond angles (^o^)	1.466
Ramachandran statistics ^e^ Favored (%)	95.38
Outliers (%)	0.29 ^f^
MolProbity score	2.00
Clashscore (all atoms)	6.48

* The high-resolution bin details are in the paratheses. ^a^ R_sym_ = ∑|Ii − <I>|/|Ii|, where Ii is the intensity of the i ^th^ measurement and <I> is the mean intensity for that reflection. ^b^ Reflections with I > σ were used in the refinement. ^c^ R_work_ = ∑|F_obs_ − F_calc_|/|F_obs_|, where F_calc_ and F_obs_ are the calculated and observed structure amplitudes, respectively. ^d^ R_free_ is the same as R_work_, but for 5–7% of the total, reflections were chosen at random and omitted from the refinement. ^e^ Ramachandran statistics were calculated using MolProbity (http://molprobity.biochem.duke.edu/ accessed on 24 October 2022). ^f^ Residue Glu64 in chain B in a tight turn, whose side chain density was not well defined.

## Data Availability

The coordinates of the *Sp*Rho1 structure in the RCSB PDB database can be found under the code 8ETD.

## Supplementary Materials

The following supporting information can be downloaded at: https://www.mdpi.com/article/10.3390/biology11111627/s1, Figure S1: Comparison of different Rho-GDP-Mg^2+^ structures. (**A**) Superimposition of *Sp*Rho1 (green) with HsRhoA (light blue, PDB code: 1FTN), HsRhoC (wheat, PDB code: 2GCN) and AfRho1 (cyan, PDB code: 5ZVP). The loop between F107 and P112, and the Rho insert domain from D125 to Q136 are highlighted with rectangles. Switch I region, Switch II region, β2, β3, the N and C termini are labeled. (**B**) The per-residue RMSD of the superimposed Rho-GDP-Mg^2+^ structures. The *Sp*Rho1 was set as a reference, for which the RMSD remains zero. Switch I, Switch II and Rho insert domain are shown with rectangles. β2 and β3 is indicated with straight line; Table S1: Hydrogen bonding contacts between the two monomers of the *Sp*Rho1 dimer; Table S2: Comparison between the *Sp*Rho1 and the other Rho subfamily proteins. References [10,11,12,15,35,36] are cited in the supplementary materials.
