# Supplementary material for: Crystal Structure of Schizosaccharomyces pombe Rho1 Reveals Its Evolutionary Relationship with Other Rho GTPases
Source: Biology (Basel). 2022 Nov 7;11(11):1627. doi: 10.3390/biology11111627 (PMC9687936; doi:10.3390/biology11111627)
Supplement: Supplementary file 1 [file biology-11-01627-s001.zip › biology-1963197-supplementary.pdf]

Supplementary Information

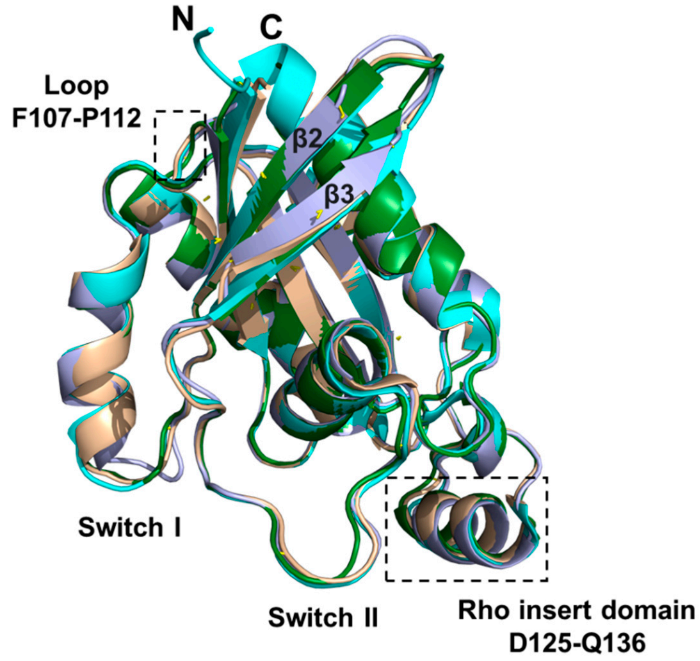

(a)

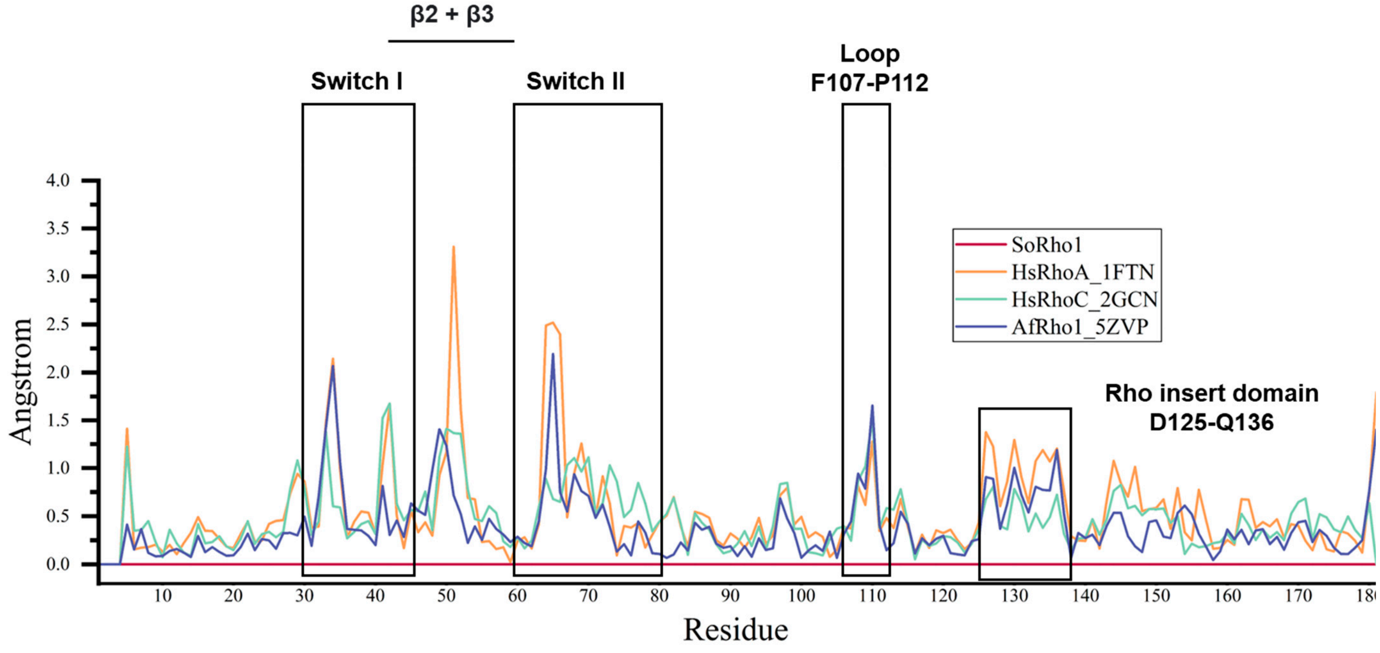

(b)

Figure S1. Comparison of different Rho-GDP-Mg^{2+} structures. **A).** Superimposition of SpRho1 (green) with HsRhoA (light blue, PDB code: 1FTN), HsRhoC (wheat, PDB code: 2GCN) and AfRho1 (cyan, PDB code: 5ZVP). The loop between F107 and P112, and the Rho insert domain from D125 to Q136 are highlighted with rectangles. Switch I region, Switch II region, β2, β3, the N and C termini are labeled. **B).** The per-residue RMSD of the superimposed Rho-GDP-Mg^{2+} structures. The SpRho1 was set as a reference, for which the RMSD remains zero. Switch I, Switch II and Rho insert domain are shown with rectangles. β2 and β3 is indicated with straight line.

Table S1. Hydrogen bonding contacts between the two monomers of the SpRho1 dimer.

Monomer A	Monomer B	Distance (Å)
Gln146 OE1	Asp50 O	3.4
Tyr157 N	Asp50 OD2	3.0
Ala154 O	Arg52 NH1	3.4
Gly153 O	Arg52 NH2	2.5
Arg52 NH2	Ala154 O	3.4
Arg177 NH1	Tyr155 O	2.9
Tyr155 OH	Tyr155 OH	2.6
Asp50 OD2	Tyr157 N	2.8
Tyr155 O	Arg177 NH1	3.2

Table S2. Comparison between the SpRho1 and the other Rho subfamily proteins.

Species	Protein name	Sequence ID (Uniprot)	Number of amino acids	Sequence Identity with *S pombe*	Structural details	Remarks
Schizosaccharomyces pombe	SpRho1	Q09914	202	100%	Present study	-
Homo sapiens	HsRhoA	P61586	193	67%	*	*
Homo sapiens	HsRhoB	P62745	196	73%	PDB 6HXU (HsRhoB Q63L-GTP-Mg^{2+}) 6SGE (HsRhoB Q63L-Nanobody B6-Mg^{2+})	Only the GTP-bound form of RhoB mediates its functions in genomic instability and in cell invasion, rather than the global expression (Bery et al. 2019 [10]).
					2FV8 (HsRhoB-GDP)	-
Homo sapiens	HsRhoC	P08134	193	70%	1Z2C (HsRhoC-MmDia-GppNHp-Mg^{2+})	HsRhoC interacts with the mDia1 at the regulatory N-terminal portion (GBD/FH3 region, an all-helical structure with armadillo repeats) (Rose et al. 2005 [35]).
					2GCN (HsRhoC-GDP-Mg^{2+}) 2GCO (HsRhoC-GppNHp-Mg^{2+}) 2GCP (HsRhoC-GTPγS-Mg^{2+})	Unlike HsRhoA which has only a single conformational transition to a signaling active state, HsRhoC undergoes at least two conformational changes as HsRhoC-GppNHp and HsRhoC-GTPγS shows differences, compared with HsRhoC-GDP, in the switch II domain and both switch I and switch II domains, respectively (Dias & Cerione 2007 [15]).
Mus musculus	MmRhoA	Q9QUI0	193	67%	4F38 (MmRho1-MmRhoGDI-GppNHp-Mg^{2+})	The membrane extraction of Rho GTPase is performed by RhoGDI via a series of

						progressively tighter intermediates. It is a passive process favored thermodynamically (Tnimov et al. 2012 [11]).
Rattus norvegicus	RnRhoA	P61589	193	67%	3TVD (RnRhoA-GTPγS-Mg^{2+})	RhoA-GTP analog complexes can be crystallized as a dimer in a centered lattice (Jobichen et al. 2012 [12]).
Saccharomyces cerevisiae	ScRho1	P06780	209	77%	3A58 (ScRho1-ScSec3N-GppNHp-Mg^{2+})	ScRho1 helps exocyst subunit Sec3 localize to the plamsa membrane (Kurokawa et al. 2010 [36]).
Aspergillus fumigatus	AfRho1	A0A068C8U8	193	81%	5ZVP (AfRho1-GDP-Mg^{2+})<br>6JIK (AfRho1-GTPγS-Mg^{2+})	-

* HsRhoA is the most well-studied Rho protein. There are 48 structures of itself (alone) or its complexes in the PDB database.
